# Pro-Inflammatory Cytokines in Nasopharyngeal Aspirate From Hospitalized Children With Respiratory Syncytial Virus Infection With or Without Rhinovirus Bronchiolitis, and Use of the Cytokines as Predictors of Illness Severity

**DOI:** 10.1097/MD.0000000000001512

**Published:** 2015-10-02

**Authors:** Patricia V. Díaz, Gonzalo Valdivia, Aldo A. Gaggero, M.R. Bono, Guillermo Zepeda, Mabel Rivas, Paola Uasapud, Ricardo A. Pinto, M. Lina Boza, Julia Guerrero

**Affiliations:** From the Institute of Biomedical Sciences, Faculty of Medicine, University of Chile (PVD, AAG, JG); Department of Public Health, Catholic University (GV); Department of Immunology, Faculty of Sciences, University of Chile (MRB); Department of Pediatrics, Faculty of Medicine, University of Chile (GZ); San Borja-Arriarán, Clinical Hospital (MR, MLB); and Family Health Center “Agustin Cruz Melo”, North Health Service, Santiago (PU).

## Abstract

Respiratory syncytial virus (RSV) and human rhinovirus (HRV) respiratory infection in children induce production of inflammatory interleukins (ILs) in the respiratory epithelium. As IL(s) determine the severity of illness, the purpose of this study was to identify the pro-inflammatory IL(s) that could be predictor(s) of clinical severity.

One hundred and fifteen patients <2 years old with bronchiolitis due to RSV and /or HRV and 38 controls were selected from a hospital and an outpatient clinic. Clinical data of all patients were recorded. Severity was defined by the number of days with oxygen need. Nasopharyngeal aspirates (NPA) were collected to perform viral diagnosis by quantitative reverse transcription and polymerase chain reaction (qRT-PCR) and to quantify ILs: TNF-α, IL-10, IL-6, IL-1β, and IL-8, by flow cytometry. Simple and multiple regression and receiver operating characteristic (ROC) curves were used for statistical analysis.

Of the patients selected 60 were single RSV, 28 RSV associated to HRV, and 27 single HRV. All patients (115) showed significantly higher IL levels when compared with controls. Levels of IL-6, IL-1β, and IL-8 detected in NPA from RSV single and associated to HRV were significantly higher than HRV infected and positively associated with days requiring O_2_.

Levels of IL-6, IL-1β, and IL-8 detected in NPA from patients infected with RSV only or with both RSV and HRV are increased, and any of those 3 cytokines may have a predictive value for the number of days with need of supplemental oxygen.

## INTRODUCTION

Respiratory syncytial virus (RSV) and more recently described human rhinovirus (HRV) are the main causes of acute respiratory tract infection (ARI) in children under 2 years of age. They may produce in previously healthy term-born infants a disease ranging in severity from mild upper respiratory infection to severe illness with bronchiolitis and pneumonia.^1–3^ The diagnosis of bronchiolitis and assessment of severity is based on history and physical examination. The severity of the disease is variable and may change in hours from a mild to a severe illness; only 2% of previously healthy infants need to be hospitalized ^4^ and there are no objective markers to predict the progress of the illness. A history of prematurity, genetic abnormalities, immunodeficiency, chronic pulmonary disease, or cardiac disease is associated with more severe disease. In full-term born infants with no previous pathology, the severity must be assessed by physical examination and sometimes prolonged observation to determine the need for hospitalization.^5^

RSV and HRV infect epithelial cells and replicate inducing numerous pro-inflammatory cytokines and chemokines including IL-12, TNF-α, IL-10, IL-6, IL-8, IL-1β, and others.^6–11^ The mediators attract cells that are predominantly inflammatory leucocytes, which increase the epithelial damage with necrosis of the epithelium lining of small airways and increased mucus production. The histopathology of fatal RSV infants shows airways obstruction related to inflammatory cell debris, mucus, and edema that explains the hypoxemia observed in more severe patients.

The majority of patients with RSV or HRV bronchiolitis show mild disease symptoms and have no need for hospitalization. However, it is sometimes difficult to predict whether they will worsen in the progression of disease. We previously ^8^ found a positive correlation between disease severity and high levels of IL-6, IL-8, and IL-1β from NPA. In this study, the primary purpose was to determine whether those proinflammatory cytokines may be utilized as an index of severity and second, whether the association of RSV and HRV would worsen the progression of the illness.

## PATIENTS AND METHODS

### Study Population

We studied 115 children <24 months of age with respiratory syncytial virus (RSV) and/or human rhinovirus (HRV) with acute bronchiolitis, and a group of 38 normal age-matched controls.

We selected patients during the RSV epidemic periods (May to September) from 2009 to 2013. Patients were attended by a pediatrician in the outpatient clinic or in the hospital. They had rhinorrhea and cough, followed by tachypnea, costal retraction, wheeze, and /or fine inspiratory crackles on auscultation. Some had a fever 24 to 72 h previously. At the time of the hospitalization, we assessed the vital signs (respiratory rate, cardiac frequency, and level of O_2_ saturation). Hospitalized children were followed until discharge to assess oxygen need (days) and length of hospitalization (days). Nonhospitalized children were examined by a doctor approximately a week later. For the purpose of this study, the assessment of severity was based on the number of days of supplemental oxygen. None of the enrolled patients required mechanical ventilation. Hospitalized patients with ≥2 days with oxygen were classified as having severe illness. Patients hospitalized with 1 or no days with oxygen and ambulatory patients were classified as having a mild infection. All patients and controls were term-born without any pathology or co-morbidities. No patients had previous significant respiratory disease or had received systemic or inhaled corticosteroids. Infants with other respiratory viruses were not included in the study. A control group selected from healthy children who underwent minor surgery with negative RT-PCR for HRV and RSV in the NPA were studied during a nonepidemic RSV period. The study was approved by the Ethics Committees of the Faculty of Medicine University of Chile, Hospital Roberto del Rio, and Hospital San Borja-Arriarán. The protocol was previously explained to parents before they signed informed consent for their child to participate in the study.

### Nasopharyngeal Aspirate Samples Collection

Nasal aspirates were taken 24 to 48 h after hospitalization or when consulting at the outpatient clinic, using a standardized technique by trained hospital personnel. A soft nasal catheter was introduced through both nostrils into the nasopharynx aspirate which was suctioned into 3 mL of sterile saline physiological solution and immediately transported on ice to the laboratory. Samples were vortexed to resolve the mucus and divided into 2 aliquots; 1 was used to study viruses RSV and HRV by qRT-PCR and the other was centrifuged to separate and identify cells, and supernatant was kept into −80°C until the cytokines and proteins were analyzed.

### Virus Studies

RSV diagnosis and patient selection were first performed by immunofluorescence assay (IFA) excluding patients with other viruses. For quantitation of RSV genomes in all patients and controls, a quantitative real-time RT-PCR was used.^12^ HRV infection was defined by positive real-time RT-PCR performed in all RSV-infected patients, in symptomatic patients with negative IFA for all viruses tested, and in controls.^13^ PCR products were purified and sequenced in both directions at Macrogen Inc (South Korea). Multiple sequence alignment was performed using the Clustal W program. Sequence alignment and phylogenetic analyses were performed with MEGA5 software,^14^ including reference sequences obtained from Gen Bank (NCBI). Sequences were assigned to the HRV genotype if they clustered with a significant bootstrap value > 50%.^15^

### Interleukins Assay

Interleukin concentrations in NPA were determined using a cytometric bead array (CBA) kit (BD-Biosciences, San Jose) in accordance with the manufacturer's protocol. We evaluated TNF-α, IL-10, IL-6, IL-1β, and IL-8, concentration by flow cytometry (FASCanto II with DIVA software, BD Biosciences, San Jose).

### Statistical Analysis

Medians were analyzed by nonparametric Kruskal–Wallis analysis of variance (ANOVA) and Mann–Whitney *U* test to compare groups, using the Graph Pad Instant program (Version 3.05 Created September 27, 2000 registered to the Universidad de Chile GTA 33483–833). *P* values inferior to 0.05 (*P* < 0.05) were accepted as statistically significant. Interleukin values were log transformed due to a non-normal distribution. Simple and multiple regression and logistic regression and odds ratio analysis were performed with days requiring O_2_ as the dependent variable, adjusting for age as the main confounder. 95% confidence interval values were also computed. Receiver operating characteristic (ROC) curves for selected interleukins were computed to search for cut-off values. Analyses were performed using STATA v.11.

## RESULTS

Table 1 shows the demographic data of the studied groups, cases versus controls, and hospitalized versus ambulatory patients. Data are expressed in means ± SD and maximum–minimum. The ambulatory patients were significantly older than the hospitalized patients (*P* < 0.001) and the control (*P* < 0.01). The higher mean and median age of ambulatory patients was calculated from 9 HRV-infected patients >12 months. The mean age of RSV patients was 5.68 months less than the HRV infected with a mean of 11.0 months (*P* < 0.001) and higher than the mean age of patients with dual infection, which was 3.92 months (*P* < 0.009).

**TABLE 1:**
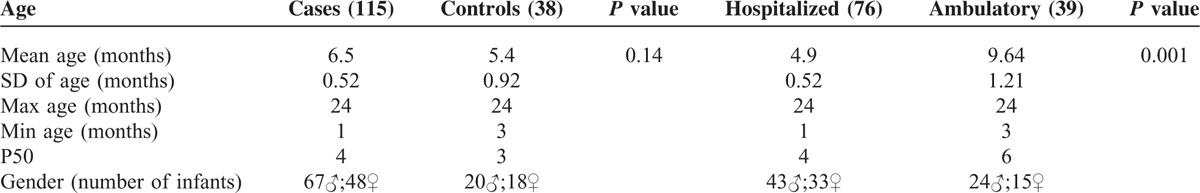
Demographic and Clinical Data

All concentrations of ILs (pg/ml) in the NPA were significantly greater (*P* < 0.01) in patients than in controls (Table 2). The ILs concentrations in NPA from patients according to the viral etiology (Table 3) IL-1β, IL-6, and IL-8 from patients with RSV were not significantly different from RSV associated with HRV, whereas levels in both groups were significantly higher (*P* < 0.01) than patients infected with HRV. TNF-α and IL-10 were not different in any group.

**TABLE 2:**
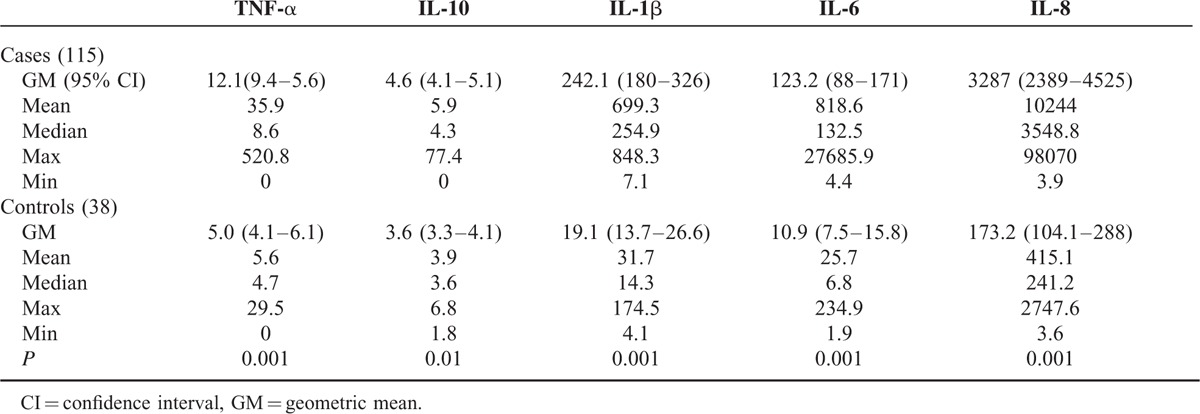
Nasopharyngeal Aspirates Interleukins Concentrations (pg/ml) as Detected from 115 Patients and 38 Healthy Controls

**TABLE 3:**
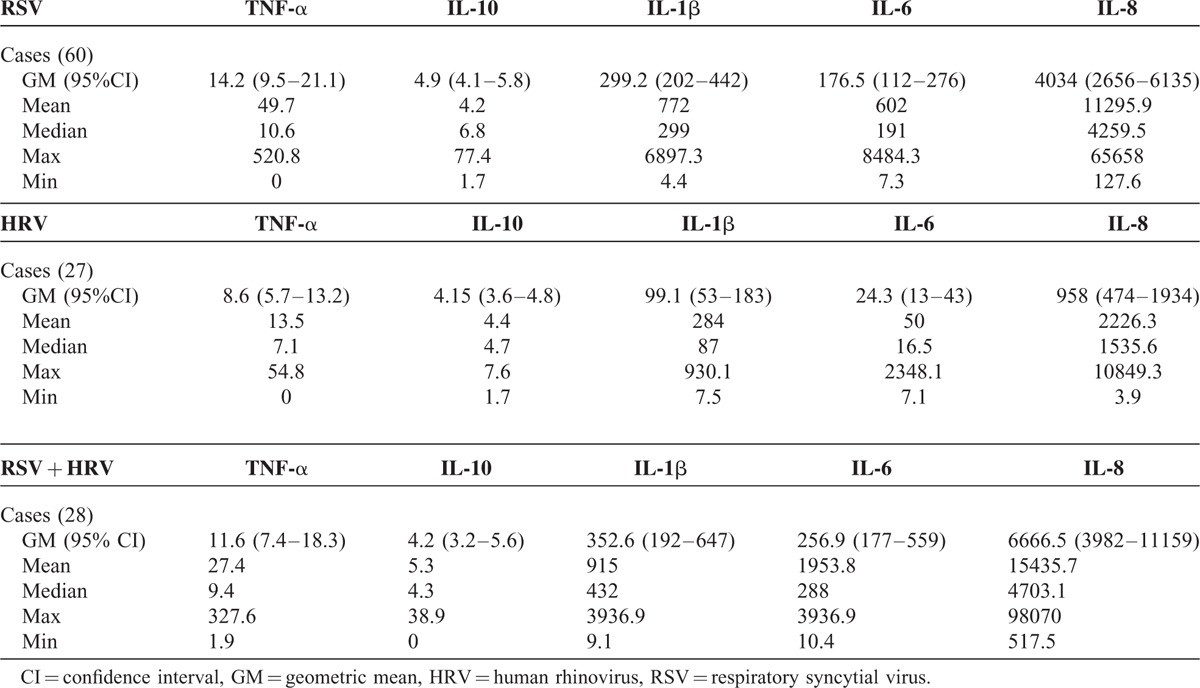
Nasopharyngeal Aspirates Interleukins Concentrations (pg/ml) From Patients With Single RSV, HRV, or dual RSV plus HRV Infections

The distribution of patients according to viral etiology and severity is shown in Table 4. Of 76 hospitalized infants, 55 (72%) needed ≥2 days of supplemental O_2_. These included 19 infants both RSV and HRV infections (34%), and 36 infants with RSV single infection (66%). HRV genotyping was performed in 30 patients; 13 of which had HRV type C, 12 were hospitalized and associated with RSV, and 1 had only HRV infection and was not hospitalized. HRV type A was found in 13 patients, 2 of which were hospitalized and associated with RSV, and in 11 ambulatory patients. In 4 patients, the HRV genotype was not determined. There was a predominance of HRV type C associated with RSV in hospitalized infants. The concentrations of IL6, IL-1β, and IL8 from severe RSV/HRV and severe single RSV infections were significantly higher (*P* < 0.01) than in infected patients with mild symptoms. There were no differences between ILs of severe RSV/HRV and severe RSV (Table 5). This is in agreement with the analysis where the odds ratio showed no risk with RSV alone versus dual infection (data not shown). Comparing the groups with mild infection, we found that only IL-6 was significantly higher in RSV patients versus HRV patients and dual (RSV/HRV) infected patients, indicating that IL-6 was related to the virus and not to the severity of infection.

**TABLE 4:**
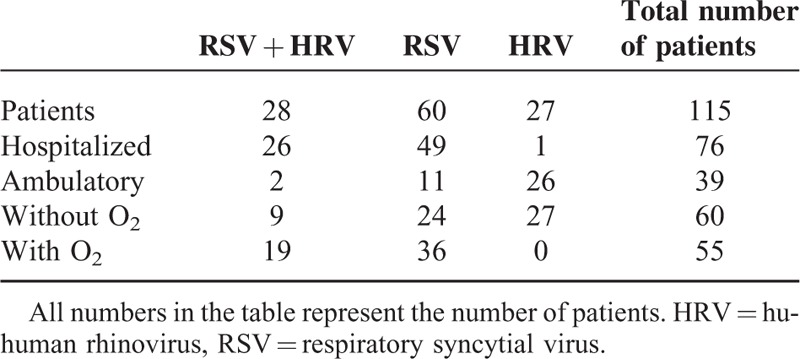
Clinical Data of 115 Patients Based on Their Viral Etiology and Needs of ≥2 Days of Supplemental Oxygen

**TABLE 5:**
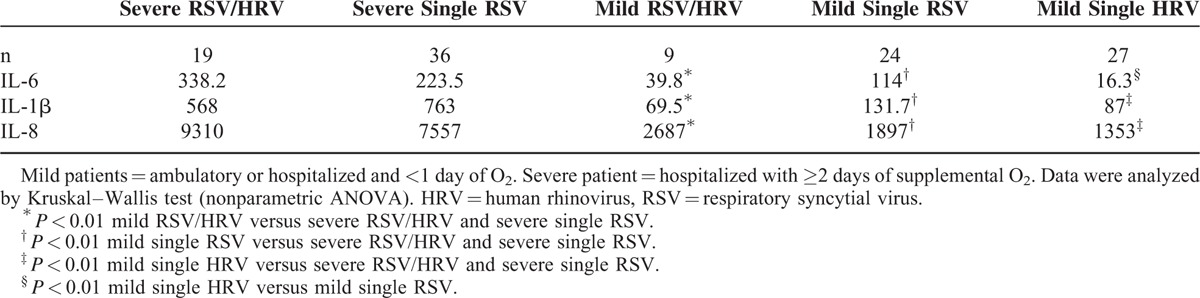
Median of IL-6, IL-1β, and IL-8 Concentrations (pg/ml) in NPA of Patients, Based on Viral Etiology and Illness Severity

A simple regression analysis performed using the number of days requiring O_2_ as the dependent variable, adjusting for age of the patients as the main confounder, shows that IL-6, IL-8, and IL-1β were significantly and positively associated with days requiring oxygen. Multiple regression analysis did not substantially change these results, indicating significant association of IL-6, IL-8, and IL-1β with this variable (data not shown).

A logistic regression analysis was used to test the dichotomous association between illness severity and viral etiology. Children with the HRV infection had a significantly lower risk of requiring O_2_ support, compared with the dual infected (RSV and HRV) group, both crude and age-adjusted analyses. An additional analysis comparing dual infection versus RSV and illness severity, excluding those children with the HRV only infection, did not show significant results.

Table 6 shows data from selected interleukins (IL-1β, IL-6, and IL-8) and sensitivity, specificity, likelihood ratios (LR), and the area under ROC curves according to illness severity, in order to determine the cut-off values and statistical differences between ROC curves. Interleukins IL-1β, IL-6, and IL-8 had high sensitivity and specificity, and IL-1β had the largest area under the ROC curves (81.2%), although there were no significant differences among the 3 ROC curves (chi^2^ −3.18, *P* = 0.204).

**TABLE 6:**
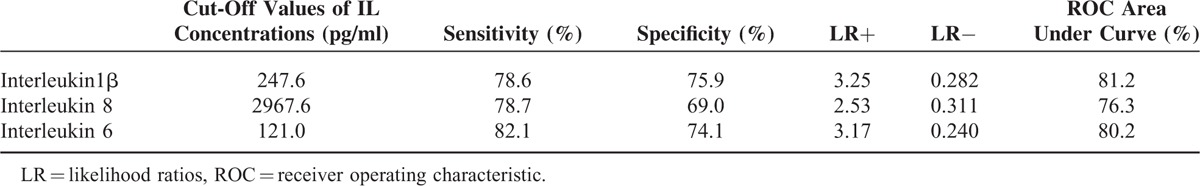
IL-1β, IL-8, and IL-6 Concentration Cut-Off Values (pg/ml), Sensitivity, Specificity, Likelihood Ratios (LR), and Area Under ROC Curves According to Illness Severity

## DISCUSSION

In this study, the diagnosis of bronchiolitis and assessment of severity was based on clinical history and physical examination performed by doctors in the outpatient clinic or the emergency room. During hospitalization, we determined the severity of the disease based on the number of days in need of supplemental O_2_ and length of stay at the hospital. The main purpose of this study is to evaluate the potential utility of a biomarker that, during the first days of the illness, would guide the decision regarding hospitalization. The candidate biomarkers proposed will require further analysis due to design limitations of the present study. Both hospitalized and nonhospitalized patients started with similar symptoms, cough, rhinorrhea, and fever and, at the time of evaluation by a doctor, wheezing, rales, inter costal retraction, and respiratory distress. The criteria used for hospitalization in this study (Chilean guidelines) were age (infants under 3 months) and oxygen saturation (<93%). It has been published that pulse oximetry at the time of decision about hospitalization does not necessarily predict the clinical outcome of number of days of hospitalization and need for oxygen ^16^ and may result in unnecessary hospitalization. Therefore, this may be the reason why in this study we found a group of hospitalized patients that needed O_2_ for only 1 day. We had previously found in a smaller number of patients that IL-6, IL-1β, and IL-8 were correlated with disease severity.^9^ In that study we used a score of illness severity previously published,^17–18^ based on the length of hospitalization, number of days with a need for supplemental oxygen, and the fraction of inspired oxygen. In this study, we analyzed the number of days of hospitalization and of need for supplemental O_2_ as separate variables. The severity among hospitalized patients in our study was not extreme, whereas other studies included more severe patients who required intensive care units.^17–18^ In this study, we may have had more hospitalized patients with mild disease due to the low socio-economic conditions of people living in the area from which the patients were selected and the Chilean guideline of bronchiolitis. The increase of inflammatory mediators during RSV and HRV respiratory infection has been extensively studied *in vivo* in nasopharyngeal samples, in *vitro* in epithelial cell culture and in blood.^6–11,19–23^ Tabarani et al^24^ found that pro-inflammatory cytokine concentrations obtained from nasopharyngeal wash was positively associated with illness severity in a larger cohort of 478 children <12 months and selected by RSV lower respiratory tract infection (LRTI), whereas in another study^18^ with a smaller number of infants, an inverse correlation with those mediators was found. We, in accordance with Tabarani et al, found a higher concentration of IL-6, IL-8, and IL-1β than IL-10 and TNF-α in NPA, and determined that the concentrations of IL-6, IL-8, and IL-1β were positively associated with illness severity. Those 3 cytokines of higher concentrations were significantly correlated with the number of days in need for supplemental oxygen, and when the results were adjusted by age as the main confounder, IL-1β, IL-6, and IL-8 remained significantly correlated with illness severity. IL-6 in serum and IL-10 in NPA have been found to correlate with illness severity.^23^ IL-1β is 1 of the 11 members of the IL-1 family and is mainly produced by alveolar macrophages and monocytes; both cell types are infected by RSV and HRV.^25–26^ IL-1β circulates systemically to the brain and induces fever but it may also be produced in hypoxemic tissues.^27^ Secretion of IL-1β may be inflammosome-dependent, by injured induced hypoventilation and hypo-perfusion.^28^ Thus, this cytokine might better reflect the need for oxygen and therefore the severity of the disease.

It is still controversial^29^ whether a single RSV infection^30^ causes less severe infection than a dual RSV/HRV infection.^31^ Our study found no risk of a more severe illness with the dual infection. However, it is interesting to note that there was a predominance of HRV type C in this study, which has been associated with more severe illness,^32^ in dual infections. Statistical issues concerning sample size and power to detect significant differences in the interleukins profile for both, RSV and HRV, were *post hoc* tested and a high power setting was concluded for this study.

(IL 1-β:89%; IL-6:98%; IL-8: 98%). A previous study^24^ testing similar research questions did not calculate the necessary sample size to test their hypotheses. This study, however, reports significant differences in the cytokines profiles.

In conclusion, quantifying these pro-inflammatory cytokines in NPA showed that IL-1β, or IL-6 or IL-8 could be a useful tool to identify those patients who will have a more prolonged disease with higher need for O_2_. This study has limitations due to the small number of patients but a biomarker such as any of those cytokines is easy to quantify and could become an easy and fast technique to help the doctor's decision.
